# Account of Remarkable Ovarian Tumors

**Published:** 1860-05

**Authors:** Joseph H. Wythes

**Affiliations:** Jeansville, Luzerne County, Penna.


ART. II.—*Account of Remarkable Ovarian Tumors.* By JOSEPH H. WYTHES, M.D., of Jeansville, Luzerne County, Penna.

So much interest has been manifested by the profession, of late years, respecting cystic tumors of the ovary, that any contribution to our knowledge respecting their nature or treatment will doubtless prove acceptable.

It has been the fortune of the writer to meet with a number of such cases, of various kinds and at different stages of development. In two or three instances, tumors, apparently ovarian, as large as a child's head, in one case persistent for four years, have been removed by the use of an ointment composed of extracts of belladonna and cicuta, with ung. hydrarg. and lard—a drachm or two of the extracts with an ounce of each of the others. In another case, where a tumor was so rapidly developed that in a month after it was noticed it had pushed aside the bladder, and acted as a mechanical obstacle to the flow of urine, no treatment I could suggest was of benefit, save for the relief of the bladder and the improvement of the general health.

Two cases were operated on, at the writer's request, by Dr. W. L. Atlee, long celebrated for his attention to this specialty, and in each case with a successful result. Both were multilocular cysts; both had extensive peritoneal adhesions, in one case rendering it necessary to divide them with the knife; and both required a long incision. The weight of one tumor was estimated at thirty pounds; the actual weight of the other, with the contained fluid, was thirty-eight pounds. One of these patients has since been delivered of a fine child, and the other enjoys excellent health.

The two cases which form the subject of the present paper present such peculiarities and suggest so much information, that an extended account of them seems due to the profession. The first is remarkable for the obscurity and complication of its symptoms and its special prolific power, and the second from the success of iodine injections after the failure of an operation for its removal.

CASE 1.—*Proliferous Ovarian Cyst simulating Artificial Anus.*—The patient was a highly intellectual young lady, aged twenty-four, who came under my care in May, 1856. I learned that about four years previously, while attempting to lift a basket of crockery, she was seized with pain in the left inguinal region, followed in a day or two by considerable swelling, which presented all the external appearances of an abscess. After some time a large amount of purulent matter was discharged by the urethra, with some relief to the patient. As the swelling still continued, and appeared to point above the pubes, it was opened at that place with a lancet. At this time she was under the hands of the homœopaths, but becoming dissatisfied, sought advice of several physicians, nearly all of whom seemed to regard it as a case of psoas abscess.

When I first saw her, I found an opening in the abdominal wall, an inch and a half above the pubes, leading into a sinus four inches long, to the left side, along the top of Poupart's ligament, from which both flatus and fæces had been passing for some time, though the most of the fæces passed naturally. On carefully dilating the entrance with sponge tents until I could pass in my forefinger, two small openings in the bowel were readily felt, about one-fourth of an inch in diameter, into each of which an elastic catheter was passed, and the angle made by them observed. It seemed to be a case of artificial anus, yet from its history I could not determine whether it proceeded from a strangulated hernia or an abscess. The circumstance of two openings existing, with the occurrence of the swelling being noticed soon after a strain in lifting, inclined me to suspect a hernia, while the occasional passage of indurated fæces indicated that the colon was involved.

I informed my patient and her family, that although compression might palliate the symptoms, an operation would be necessary; yet as she was considerably emaciated, and warm weather was approaching, I deemed it advisable to premise such means as would recruit her general health, with a visit to the sea-side, etc.

When cold weather approached, she became anxious for relief, and I called a neighboring physician in consultation, who urged a continued trial of compression before resorting to the knife. At this time we found but one opening in the bowel, large enough to admit the end of the little finger, although a pit or depression existed in the sinus at the place of the other opening.

Compression had no further effect than to prevent the escape of fæces quite so readily. Her general health also suffered, and she became urgent for an operation. I therefore invited Professor Gross, of the Jefferson Medical College, to visit the case, April 9th, 1857. Concurring in the necessity of an operation, in the presence of Drs. Carpenter, Rhein, and the writer, he laid open the sinus, the patient being etherized. A large amount of unorganizable lymph was removed, but the opening in the bowel was found to be single, and so small as to afford a prospect of cure without resorting to a suture. A roll of lint was placed in the cavity and covered with a compress, and the patient directed to remain quiet.

For some days the bowels were kept at rest by the use of elixir opii, and the dressings removed from time to time. The opening in the bowel contracted somewhat, but although every means was taken to improve her general health, it did not heal, nor would the sinus fully granulate. In the beginning of October, I applied an elastic bandage over the abdomen and allowed her to walk about, and by the tenth of November the sinus had entirely healed. On the 30th of November, however, a small opening occurred, giving exit to a little pus, and in a day or two after another opening was seen. These were small sacs or pockets in the course of the original sinus, the old opening into the bowel remaining firm. As they appeared of but trifling extent, they did not prevent the patient from exercise; yet I deemed it prudent to prescribe occasional tonics.

As my patient was of singular benevolence of heart, on February twenty-fifth she visited a poor man who lay ill of the gravest form of typhoid fever, and on the two succeeding days used excessive exertion on the occasion of the funeral of a friend. On the morning of the twenty-eighth I was sent for, and found her much prostrated, with nervous excitability and jactitation; pulse 120, and tongue covered with a whitish fur. Prescribed a calmative mixture, and a dose of castor-oil and laudanum to be taken in the evening. I saw her again at night, and, as the oil had operated, gave a full dose of Dover's powder. The next day she complained of pain when pressed over the cæcum; there was also slight tympanitis, with thirst and a brown tongue. Although her intellect remained clear, the symptoms were so similar to those of ordinary nervous or typhoid fever, that I had no difficulty in diagnosis; yet from her previous condition I feared a speedy perforation of the bowel. Unfortunately, my fears were too well founded, as about 9 A.M., on the 4th of March, I saw her breathe her last.

A post-mortem examination having been permitted by the family, in the presence of Dr. Rhein, and my student, Mr. Dimmick, I next day made a thorough investigation of the condition of the thoracic and abdominal viscera. An examination of the old sinus was made, and no communication could be found with the internal viscera. The state of the muscles was learned by careful dissection, and they were observed to be so fused together as to make that part of the abdominal wall really stronger than elsewhere. On opening the cavity of the peritoneum it was found full of feculent matter, apparently from a perforated bowel. The intestines were much softened, although adherent to each other, and to the omentum and peritoneum, by old and strong attachments. Indeed, every organ examined, lungs, pleura, stomach, liver, etc., were adherent in the most remarkable manner. From the effort necessary to detach the intestines, and their softened condition, it was impracticable to find the precise point of perforation. With the exception of the adhesions referred to, the lungs were healthy. They were particularly examined for tubercular deposit. The heart and pericardium were healthy, and were the only structures observed without abnormal adhesions.

Immediately in front of the bladder was an ovarian tumor or cyst, oval in shape, about five inches long and four broad. On slitting it lengthwise, a large mass of sebaceous matter was seen, full of hair, and two irregular bones were observed in the membranous wall, into which eleven well-formed teeth were inserted. One of these pieces had two rows of teeth, some molar, others irregular in shape. This cyst had been the origin of all the former difficulty, as it was found adherent to the descending colon and peritoneum in the neighborhood of the old sinus, as well as to the fundus of the bladder, which was torn in the effort to detach it. The descending colon was considerably diminished in calibre, and had been perforated from side to side. The uterus had been so pressed upon by the tumor as to be flattened, and distorted into an anteflexion. The right ovary appeared healthy, saving a round, hard, ivory-like concretion, the size of a pea.

This tumor had so displaced the organs, that in connection with a remarkably capacious pelvis, it was not possible to ascertain its presence by external or tactile investigation.

The pieces of bone in the tumor were covered with membrane presenting all the characteristics of true skin, from which proceeded the hair before alluded to. Under the microscope, this hair presented nothing remarkable, save the comparative absence of pigment, and a sharply serrated appearance of the outer imbricated scales. The fibres, as might be expected, seemed more loosely bound together than in other hair. The amount of hair, when washed from sebaceous matter, was about a large handful.

Instances of cutaneous proliferous cysts are given by Mr. Paget, in his Surgical Pathology, as occurring in the subcutaneous tissue, in the brain, in the umbilicus, and in the scalp, and teeth-bearing cysts in the jaws, particularly in the antrum. One case is recorded in which the tumor was found in the anterior mediastinum of a woman twenty-one years of age, and contained skin and fat, serous fluid, sebaceous matter, and two pieces of bone, like parts of upper jaws, in which seven well-formed teeth were imbedded. Four similar cases occurring in the ovary are on record. Two from the museum of the Royal College of Surgeons, and two from an essay by Lang. In one of Lang's cases more than three hundred teeth were found in the ovary; in the other, a piece of bone, like part of an upper jaw, with forty-four teeth. "It is only," says Mr. Paget, "during the vigor of the formative forces in the fœtal or earliest extra-uterine life, that cysts thus highly organized and productive are ever formed." The history of this case, however, causes us to entertain a doubt upon the correctness of this opinion. From this it appears that she first menstruated when between fifteen and sixteen years of age, and that she imprudently washed all over in cold water, which produced illness, rendering medical treatment necessary. Although relieved, she had no further menstrual discharge till she was seventeen years old; yet during this time such was the vigor of the vital forces that she grew nine inches in height in three months time, as ascertained by measurement. This fact, which is well authenticated, points out that period as the time, in all probability, of the formation of the cyst. When between seventeen and eighteen years old a peculiar fetor of the breath was observed, which continued until the discharge by the urethra, and was afterwards always noticed when no discharge occurred.

CASE 2.—*Ovarian Cyst cured by Iodine Injections after an unsuccessful Attempt at Removal.*—On May 16, 1858, I was consulted by Bridget McK., aged twenty-two. She was always regular till after an attack of fever, one year and nine months ago, when she noticed a swelling in the right iliac region, which steadily increased. Has had no menstrual discharge since. Her abdomen was as large as at full term of gestation; the umbilicus protruded, and there was evident fluctuation. The womb was small and retroverted, but appeared movable, and the sound entered one and a quarter inches. Abdomen so tense that I could not judge as to adhesions.

I informed her that she had ovarian dropsy, and explained the risks of the operation to which she was anxious to submit. Her general health and spirits were so good that I anticipated a favorable issue.

I tapped her on May twenty-fifth, in the presence of Drs. Brown and Nicholas, and my student, Mr. Dimmick, of five gallons of fluid, yellow, and semi-opake, strongly albuminous on boiling, and in which the microscope showed short hairs or acicular crystals, cholesterine plates, and compound characteristic granules.

In two days after tapping, the cyst appeared to fill rapidly, and was most evident on the right side, seeming to be quite loose in the peritoneal cavity, from which I was led to suppose the absence of important adhesions.

She went home, and returned on the thirty-first to be operated on. Her abdomen was quite full and tense, and measured forty-six and a half inches in circumference. A full dose of castor-oil was given, with a little laudanum, to prepare her for the operation. This not answering the purpose, I repeated it on June first.

The operation took place, on the 2d of June, in the presence of a large number of medical gentlemen. Before proceeding, I noticed a subsidence in the size of the tumor, which I mentioned to the gentlemen present, together with the mode of arriving at the diagnosis, and proposed to make an explorative incision, and, if possible, extract the sac.

After anæsthesia had been produced by a mixture of chloroform and ether, I made an incision four inches long, in the course of the linea alba. From the flaccid condition of the abdominal parietes it was necessary to divide the layers with a bistoury upon a grooved director. Upon reaching the peritoneum, which was altered in character, thick, fibrous, and fused, as it were, into the adjoining tissues, a fold of it was raised with a tenaculum, and a small incision made for the purpose of introducing the director, when a sudden gush of fluid showed that I had punctured the sac. The fluid was allowed to drain off, and the sac carefully explored. It was found universally adherent to the abdominal parietes and viscera. I made an attempt to break up the adhesions on one side with my finger, but finding them firm, abandoned all hope of removing the cyst. A tent was introduced into the lower angle of the wound, the edges of the incision were brought together by four twisted sutures and strips of adhesive plaster, a wet compress and bandage placed over all, and the patient put to bed.

The after-treatment consisted of strict antiphlogistic regimen and opiates. As the patient was troubled with bilious vomiting, and the pulse kept excitable, I did not resort to the iodine injections until the 15th of June, when, after evacuating a pint and a half of exceedingly offensive pus, I passed through a flexible catheter two ounces of tincture of iodine. No irritation whatever having been produced, on the seventeenth I injected four ounces more. On the day following, I found the contained fluids so coagulated that the mass was with difficulty broken up by the catheter. I allowed several days for its removal, and having washed the sac well with tepid water, injected three ounces more, which was the exact capacity of the sac. I continued the injections every few days until the middle of July, using in all about a pint and a half of tincture, when the sac had contracted so much as to admit but about an inch of the catheter. An elastic abdominal bandage was put on and the patient allowed to return home. I have seen her at various intervals since up to the present time. There has been no return whatever of the enlargement, although as a measure of precaution she continues the bandage. Her health has been fully re-establislied, and her menses returned five months ago, and have since been regular.

